# Mechanically-induced osteogenesis in the cortical bone of pre- to peripubertal stage and peri- to postpubertal stage mice

**DOI:** 10.1186/1749-799X-4-22

**Published:** 2009-06-25

**Authors:** Jeffrey H Plochocki

**Affiliations:** 1 Department of Anatomy, Midwestern University, Glendale, Arizona, USA; 2 Department of Biology, The Pennsylvania State University, Altoona, Pennsylvania, USA

## Abstract

**Background:**

Exercise during postnatal development plays a key role in determining adult bone mass and reducing the risk of fracture and osteoporosis later in life. However, the relationship between mechanically-induced osteogenesis and age is unclear. Elevated levels of estrogen during puberty may inhibit periosteal bone formation. Thus, magnitudes of mechanically-induced osteogenesis may be vary with pubertal state.

**Methods:**

The present study uses a murine model to examine age-related changes in bone formation at the femoral midshaft with voluntary exercise. Pre- to peripubertal mice aged 3 weeks and peri- to postpubertal mice aged 7 weeks were randomly divided into sedentary and exercised groups and subjected to histomorphometric comparison after 4 weeks of treatment.

**Results:**

Results of the experiment indicate that exercise significantly increased osteogenesis on the periosteal and endocortical surface of the mice in the older age group (*P *< 0.05). Exercise had no significant effect on bone formation of mice in the younger age group, although exercised mice exhibited more bone growth on average than controls. Endocortical apposition was the primary method of bone formation for all mice in the experiment; however exercised mice in the older age group were able to add more bone on the periosteal surface than age-matched controls and exercised mice in the younger age group (*P *< 0.05). Medullary area increased with age, but exercised mice in both age groups had smaller medullary cavities relative to overall bone area than controls.

**Conclusion:**

These findings suggest that the amount and location of mechanically-induced osteogenesis differs by age during skeletal development. Late adolescence may be the optimal time to accrue bone mass and maximize bone strength.

## Introduction

Loss of bone mass is a serious concern for the aging population. Older individuals affected by osteopenia and osteoporosis are at greater risk for age-related fractures, which are associated with increased morbidity and mortality [1]. Emerging evidence suggests that peak bone mass is closely related to the severity of bone loss later in life [2]. Peak bone mass is determined by both genetic and environmental factors, including the mechanical environment of the skeleton during postnatal development [3,4]. Physical inactivity in subadults is associated with lower bone density, and hence a greater risk of fracture, than those who are more physically active [5]. Thus, physical activity levels during skeletal development regulate osteogenesis to affect bone mass and fracture risk later in life [4].

It is clear that alterations in the magnitude of mechanical loading of the skeleton can directly influence bone formation and maintenance during periods of skeletal growth. However, the relationship between osteogenic response to exercise and age is less clear. Bone formation on the periosteal and endocortical surfaces is regulated, in part, by hormones like estrogen [6]. Estrogens have an inhibitory effect on periosteal apposition and endocortical resorption [7,8]. Since hormone levels change throughout development, their effect may vary with age [9,10]. The addition of bone on the periosteal surface provides greater resistance to bending than endocortical bone formation because it adds bone further from the bending axis. Periosteal apposition would then be the optimal response to increased mechanical loading because it maximizes bone strength. However, elevated estrogen levels during puberty may inhibit periosteal bone formation, thereby promoting exercise-induced osteogenesis on the endocortical surface. Therefore, there may be an age at which exercise more effectively increases bone strength while adding bone mass. Such information is important to clinicians for understanding determinants of peak bone mass and bone strength, and to assess fracture risk later in life.

Previous investigations into the relationship between mechanical and hormonal regulation of diaphyseal growth have yielded mixed results [8,10,11]. Mechanical loading may accelerate periosteal growth before puberty and endocortical expansion in the postpubertal stage [8,10], but the effects of loading and estrogen may be independent of each other [11]. The aim of this study is to further examine the relationship between age and mechanically-induced bone formation from voluntary exercise in laboratory mice. Voluntary exercise treatment is used because it allows for activity levels and durations within a normal physiological range. Bone histomorphometric comparisons are made between prepubertal to peripubertal and peripubertal to postpubertal mice to test the hypothesis that the location of bone formation on the femoral diaphysis differs with age, and consequently internal hormonal environment.

## Methods

Forty-two virgin female mice of the strain C57BL/6J were used in the experiment (Jackson Laboratory, Bar Harbor, ME). Mice were housed in 153 in^2 ^cages and provided with food and water *ad libitum*. After a one week acclimatization period, the mice were divided into four groups: 11 mice aged 3 weeks treated with exercise, 11 sedentary control mice aged 3 weeks, 10 mice aged 7 weeks treated with exercise, and 10 sedentary control mice aged 7 weeks. These ages were chosen because puberty in C57BL6 mice typically occurs by the 6^th ^or 7^th ^week of life and ends shortly thereafter [12]. The 3-week-old mice in this experiment are prepubertal to peripubertal, while the 7-week-old mice are peripubertal to postpubertal. Exercise treatment involved continuous voluntary access to an activity wheel (Bio-Serv, Frenchtown, NJ). Use of the wheels was monitored by magnetic counters and regular periods of observation. All mice received an intraperitoneal injection of calcein (Sigma, St. Louis, MO) at a dose of 30 mg/kg of body mass on day 8 and 22 of the experiment. The calcein acts as a fluorochrome label to identify areas of active bone formation.

The experiment lasted for 4 weeks, after which the mice were sacrificed with compressed carbon dioxide at 7 and 11 weeks of age respectively. Thus, mice in the younger age group were treated between the ages of 3 to 7 weeks, while mice in the older group were treated from 7 to 11 weeks of age. Note that skeletal growth is typically negligible after the 16^th ^week of life in this strain of mice [13]. Femora were harvested and cleaned of soft tissue and then secured in 10% NBF, dehydrated in alcohol, and imbedded in methyl methacrylate (Polysciences, Warrington, PA) for sectioning. A low speed saw (Isomet; Buehler, Lake Bluff, IL) was used to section the femora in the transverse plane at midshaft. Sections were ground to a final thickness of roughly 20 μm for analysis. Digital captures of the sections were taken under fluorescent microscopy and histomorphometric data was recorded using ImageJ 1.40 g . Histomorphometric parameters were obtained and bone formation rates were calculated using the calcein labels. Endosteal and periosteal perimeters were measured as the curved length of the endosteal and periosteal surfaces. Medullary area was calculated as the area of the medullary cavity. Periosteal area was calculated as cortical area + medullary area. Areas of endocortical and periosteal bone growth were measured as the areas of new bone on the endosteal and periosteal surfaces respectively as indicated by the fluorochrome labeling. Total growth area was calculated as the area of bone growth on the periosteal surface + the area of endocortical bone growth. Bone formation rate was calculated as the area of bone added per day at the endosteal and periosteal surfaces. Differences between variables were tested using analysis of variance (ANOVA). A two-way ANOVA with age and exercise treatment as the main effects was used to test for age-exercise treatment interactions. Significance was set at *P *< 0.05.

## Results

During the 4-week experiment, exercise-treated mice ran an average of 8.3 km per day with a standard deviation of 1.08 km (7-week-old mice: 8.33 km/day, 1.10 S.D.; 11-week-old mice: 8.27 km/day, 1.05 S.D.). There was no significant difference in running distance between exercise-treated mice in the two age groups (*P *> 0.05). However, voluntary running exercise had a significant effect on body mass. Exercised mice in both age groups had greater body mass than controls at the end of the experiment (*P *< 0.05), although no significant difference in body mass existed at the beginning of the experiment.

Table 1 displays summary statistics of the histomorphometric parameters of the femoral midshaft and the results of the ANOVA. The analysis indicated there are significant differences in histomorphometric parameters between age groups. Eleven-week-old mice in the exercise-treated group had significantly larger endosteal and periosteal perimeters, and cortical, medullary, and periosteal areas than exercise-treated mice in the 7 week age group (*P *< 0.05). Similarly, control mice in the 11 week age group had significantly larger endosteal and periosteal perimeters, and cortical, medullary, and periosteal areas than their counterparts in the 7 week age group (*P *< 0.05). Age group had no effect on the rate and area of bone growth, except at the periosteal surface. No significant differences were found in the area of endocortical bone growth, area of total bone growth, and bone formation rate by age group. Exercised 11-week-old mice, however, had a significantly greater area of periosteal bone growth than 7-week-old exercised mice (*P *< 0.05).

**Table 1:** Means and standard deviation of femoral parameters in control and exercise-treated mice of both age groups

	7-week mice		11-week mice	
	
	Control	Exercised	Control	Exercised
Endosteal perimeter (mm)	3.64 (0.14)	3.73 (0.16)	3.73 (0.15)^a^	3.85 (0.32)^a^
Periosteal perimeter (mm)	2.02 (0.18)	2.03 (0.13)	4.69 (0.14)^a^	4.83 (0.27)^a, b^
Cortical area (mm^2^)	0.50 (0.15)	0.52 (0.09)	0.55 (0.04)^a^	0.61 (0.04)^a^
Medullary area (mm^2^)	0.31 (0.05)	0.32 (0.04)	0.93 (0.07)^a^	0.98 (0.14)^a^
Periosteal area (mm^2^)	0.81 (0.15)	0.84 (0.08)	1.49 (0.10)^a^	1.59 (0.17)^a, b^
Area of endocortical bone growth (μm^2^)	26.50 (2.41)	44.66 (3.48)	35.60 (1.13)	51.20 (1.68)^b^
Area of periosteal bone growth (μm^2^)	9.37 (0.28)	9.94 (0.19)	7.30 (0.68)	16.20 (0.62)^a, b, c^
Total bone growth area (μm^2^)	35.87 (2.38)	54.59 (3.37)	42.90 (1.46)	67.40 (1.55)^b^
Bone formation rate (μm^2^/day)	2.56 (1.69)	3.91 (2.41)	3.06 (1.04)	4.81 (1.11)^b^

^a ^Significant difference between age groups by exercise treatment group

^b ^Significant difference between exercise treatment groups by age group

^c ^Significant age group and exercise treatment group interaction

**Figure 1:**
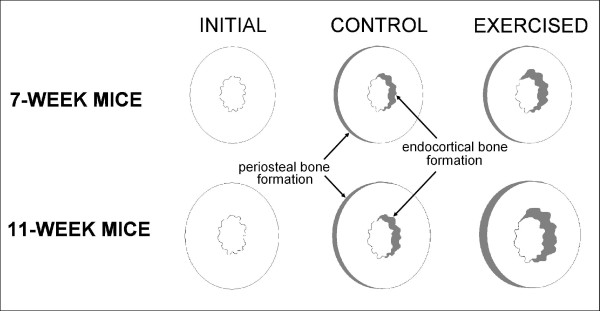
**Schematic representation of changes at the femoral midshaft with variable exercise treatment of mice aged 7 and 11 weeks**. Shaded regions indicate areas of bone growth. Cortical areas of 7-week-old mice treated with voluntary running exercise were ~4% greater than age-matched controls, although this difference was not significant (*P *> 0.05). Cortical areas of exercise-treated mice aged 11 weeks was ~10% greater compared with controls (*P *< 0.05). Mice in both age groups exhibited about 3.8 times more endocortical growth than periosteal growth in a pattern indicative of anterior diaphyseal drift (anterior is to the left in this image).

The primary location of bone growth in all groups was on the endosteal surface. On average, endocortical bone growth exceeded growth on the periosteal surface by a factor of 3.8 in both exercised and sedentary control mice of both age groups. The effect of exercise treatment varied with age (Figure 1). No significant histomorphometric differences were found between exercised and sedentary control mice in the 7 week age group (Table 1). However, exercised 11-week-old mice had significantly greater cortical area, areas of endocortical and periosteal bone formation, and bone formation rate in comparison to controls (*P *< 0.05). Only one variable, area of periosteal growth, yielded a significant interaction between age group and exercise treatment with the two-way ANOVA (*P *< 0.05). Exercised mice aged 11 weeks showed significantly greater periosteal growth in comparison to sedentary controls than similarly treated mice in the 7 week age group.

**Figure 2:**
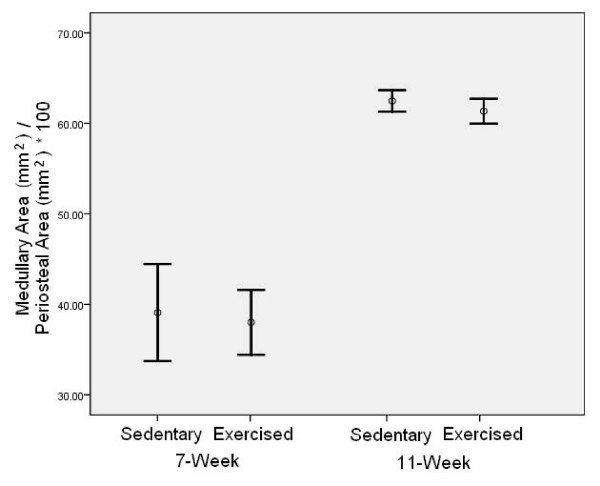
**Error bar pots (mean ± 2 SE) of medullary area expressed as a percentage of periosteal area for exercised and sedentary mice aged 7 and 11 weeks**. No significant differences exist between exercised and sedentary mice of either age group. However, mice in the older group have substantially larger medullary areas for their given periosteal area (*P *< 0.05).

To assess relative size differences at the femoral midshaft with exercise and age, a comparison of the ratio of medullary area to periosteal area was made (Figure 2). This statistic gives the percentage of the medullary area to the total area of space within the periosteal perimeter. Although no differences were found between exercise and control mice within each age group, a significant difference was found between age groups. Mice in the 11 week age group have substantially larger medullary areas for their given periosteal area than mice in the 7 week age group (*P *< 0.05).

## Discussion

Diaphyses of load-bearing bones of the lower limb are the most susceptible to fracture in individuals with low bone mass [14] and thus a concern to the aging population. The skeleton has the greatest capacity to add bone mass in response to exercise before skeletal maturity is reached [15,16], but the precise relationship between age and the site of bone formation in the lower limb remains unclear. Using a mouse model, our data indicate that endocortical apposition was the primary method of bone formation in both age and treatment groups. Despite this, medullary and cortical area enlarged as age increased, suggesting endocortical resorption is taking place simultaneously with endocortical apposition, possibly as part of diaphyseal drift (Figure 1). Similar results have been reported using animal exercise models [17,18]. This pattern of bone formation would result in a greater concentration of mass further from the geometric centroid of the bone cross-section to maintain resistance to bending during growth as body mass and femur length increase [19]. Exercised mice in the older age group also had greater bone growth on the periosteal surface than controls. Again, this growth pattern would serve to increase the ability of the bone to resist bending forces in exercised mice relative to controls because there is more bone mass further from the bending axis to resist strain for any given bending moment [20].

The magnitude of endocortical and periosteal bone growth and rate of bone formation showed little change with age. The average bone formation rate and area of bone growth did not differ significantly between pre- to peripubertal and peri- to postpubertal mice in either treatment group. Given that strain rates are likely comparable within treatment groups regardless of age, similar growth rates may be expected [21]. However, there was a significant age-exercise interaction with the area of periosteal bone growth, but not endocortical growth. The dissimilarity in the location of osteogenesis in response to loading in the two age groups suggests periosteal bone formation is regulated differently at these pubertal stages, as hypothesized. Because estrogen inhibits periosteal bone apposition and endosteal resorption [22,23], mechanically-induced osteogenesis may be limited at the periosteal surface around 6 to 7 weeks of age in mice. This would explain why mice in the older age group exhibit a greater osteogenic response to exercise but also greater endosteal resorption as indicated by their larger medullary cavities.

It should be noted that the findings reported here are not entirely consistent with Garn's model based on observation of the metacarpal [24] that proposes growth occurs at both the periosteal and endocortical surface in prepubertal females, but periosteal apposition is reduced in pubertal females. The results of this study suggest exercise does not simply exaggerate the growth pattern described by Garn, but rather acts in a regulatory role in conjunction with estrogen to differentially affect the location of osteogenesis. These data are more consistent with those of Bass et al.[10] from the humeral midshaft in human tennis players. Using MRI, they report periosteal apposition and endocortical resorption without endocortical apposition in peripubertal females, with increases in periosteal apposition in postpubertal females. The results presented here experimentally confirm this finding in mice, but fluorochrome labels also indicate that, despite a net increase in the size of the medullary cavity in the younger age groups, endocortical apposition occurs as well. Shi et al. [25] also found that late adolescent schoolchildren are better able to increase long bone mass than younger children in response to exercise. Our data support this finding and further suggest that cortical mass is added on the periosteal surface at a faster rate than endocortical surface following increased mechanical loading within the normal physiological range.

In light of the findings of the current study, it is clear that the relationship between age and mechanically-induced bone formation is complex and dynamic. Mice in the peri- to postpubertal age group demonstrated a greater osteogenic response to increases in mechanical loading than the pre- to peripubertal mice. All mice in the experiment exhibited more growth on the endocortical surface than the periosteal surface, but periosteal apposition is responsive to mechanical loading during later growth. The results suggest that the period of optimal bone mass accrual may occur during peri- to postpubertal growth, which would translate to late adolescence in humans. Activity during this time period may be particularly important for adding bone mass and minimizing the risk of bone loss and fracture in adulthood. Clearly the effects of age on the location and rate of mechanically-induced bone formation are not simple. Currently, there are no detailed time course change data on bone histomorphometric parameters from prepubertal to postpubertal stages. More research is needed to better understand the complex relationship between the hormonal regulation of bone formation and the mechanical environment.

## Competing interests

The author declares that they have no competing interests.

## Authors' contributions

JHP conceived and performed the study.
